# Free-Standing Carbon Nanofiber Films with Supported Cobalt Phosphide Nanoparticles as Cathodes for Hydrogen Evolution Reaction in a Microbial Electrolysis Cell

**DOI:** 10.3390/nano14221849

**Published:** 2024-11-19

**Authors:** Gerard Pérez-Pi, Jorge Luque-Rueda, Pau Bosch-Jimenez, Eduard Borràs Camps, Sandra Martínez-Crespiera

**Affiliations:** 1 Applied Chemistry and Materials Department, Leitat Technological Centre, C/Innovació, 2, 08225 Terrassa, Spain; geperez@leitat.org; 2 Circular Economy & Decarbonization Department, Leitat Technological Centre, C/Innovació, 2, 08225 Terrassa, Spain; jluque@leitat.org (J.L.-R.); pbosch@leitat.org (P.B.-J.); eborras@leitat.org (E.B.C.)

**Keywords:** electrospinning, electrocatalyst, cobalt phosphide, carbon nanofiber (CNF), nanoparticles, free-standing films, cathodes, hydrogen evolution reaction (HER), microbial electrolysis cells (MECs)

## Abstract

High-performance and cost-efficient electrocatalysts and electrodes are needed to improve the hydrogen evolution reaction (HER) for the hydrogen (H_2_) generation in electrolysers, including microbial electrolysis cells (MECs). In this study, free-standing carbon nanofiber (CNF) films with supported cobalt phosphide nanoparticles have been prepared by means of an up-scalable electrospinning process followed by a thermal treatment under controlled conditions. The produced cobalt phosphide-supported CNF films show to be nanoporous (pore volume up to 0.33 cm^3^ g^−1^) with a high surface area (up to 502 m^2^ g^−1^) and with a suitable catalyst mass loading (up to 0.49 mg cm^−2^). Values of overpotential less than 140 mV at 10 mA cm^−2^ have been reached for the HER in alkaline media (1 M KOH), which demonstrates a high activity. The high electrical conductivity together with the mechanical stability of the free-standing CNF films allowed their direct use as cathodes in a MEC reactor, resulting in an exceptionally low voltage operation (0.75 V) with a current density demand of 5.4 A m^−2^. This enabled the production of H_2_ with an energy consumption below 30 kWh kg^−1^ H_2_, which is highly efficient.

## 1. Introduction

The energy sector is responsible for more than 70% of global GHG emissions related to global warming and climate change. Achieving climate neutrality by 2050 (European Green Deal) requires us to substantially reduce the use of fossil fuels by replacing them with renewable energy sources. In this sense, H_2_ represents a potential energy vector capable of addressing the variable generation profile of the renewable energies [1]. An example of this importance is the REPowerEU Plan [2], in which the EU Commission has the objective of using 20 million tons of green H_2_ in 2030 (10 Tm produced in EU). This means the installation of 80–100 GW of electrolyser capacity, which is one of the most promising technologies to produce H_2_, using electrolytic water splitting [3].

There are several types of electrolysis technologies with different states of maturity and key indicator operation parameters [4]. Examples of mature devices at industrial and commercial scale are the alkaline (AEL) and proton exchange membrane electrolysers (PEMEL), while the solid oxide (SOEL) and anion exchange membrane electrolysers (AEMEL) are under development. However, currently commercial electrolysers produce H_2_ at too high costs (>3.5 € kg H_2_), have limited efficiencies (>55 kWh kg^−1^ H_2_), and have limited industrial production capacity. To overcome both challenges, it is important to diversify and develop improved electrolyser technologies and components.

Microbial electrolysis cells (MECs) are a type of bioelectrochemical system used for H_2_ production. Within a MEC system, electrochemically active bacteria perform the oxidation of organic matter, yielding electrons and protons. These bacteria transfer the electrons to the anode, while the protons are released into the bulk solution. Subsequently, the electrons travel through an external conductive wire to the cathode chamber, where they perform the HER, producing H_2_ [5,6]. The MEC systems were discovered in 2005, and they received attention for their decrease in required cell voltage due to the anode reaction from the oxidation of organic matter (E^’^_0_ = −0.28 V vs. SHE) instead of an oxygen evolution reaction (OER) (E^’^_0_ = 0.82 V vs. SHE). Considering the HER at the cathode (E^’^_0_ = −0.41 V vs. SHE), the resulting theoretical cell voltage of the MEC systems is significantly lower than in electrolysers (−0.13 V vs. 1.23 V, respectively) [7]. According to this, the overpotentials of the MEC systems are operated between 0.7 and 1.5 V and the theoretically H_2_ production required energy is between 20 and 40 kWh kg^−1^ H_2_, which is significantly lower than for AEL, PEMEL, SOEL, or AEMEL [8]. However, MEC systems have lower current density (1–15 A m^−2^) compared to AEL and PEMEL (6000–20,000 A m^−2^), which limits the H_2_ production rate. Although MEC systems have good results in terms of H_2_ production at laboratory scale, showing rates between 1 and 10 m^3^ H_2_ m^−3^ reactor and day^−1^, the main challenge is achieving the same results with industrial-relevant reactors working with real wastewater. Up to now, the studies with up-scaled MEC reactors reported production rates below 0.1 m^3^ H_2_ m^−3^ reactor and day^−1^ [9]. Despite these challenges, MEC reactors work well with wastewater at the anode, avoiding the requirement to use high-purity water and allowing the degradation of pollutants (mainly organic matter) contained in the wastewater. Thus, MEC systems allow the treatment and valorization of wastewater to produce renewable H_2_.

MEC reactors can work with an alkaline catholyte and, therefore, do not require platinum group metal (PGM) electrocatalysts, as in the case of AEL and AEMEL. This is advantageous since PGMs, such as Pt, IrO_2_, and RuO_2_, that are best used as electrocatalysts have a high cost and are scarce [10]. Hence, the research of abundant and high-performance catalysts with long-term stability becomes a crucial step for producing H_2_ from electrocatalytic water splitting.

To date, many kinds of non-noble metal composites, including metal alloys, oxides, hydroxides, chalcogenides (selenides, sulphides), carbides, nitrides, borides, and phosphides, have been investigated and identified as active catalysts in electrocatalytic water splitting [11,12]. Among them, transition metal phosphides (TMPs) have shown excellent intrinsic activity and unique electrochemical properties [13,14,15]. The incorporation of electronegatively charged phosphorous (P) atoms into the metal lattice results in negatively charged P atoms with positively charged metal atoms that accept protons and hydrides, respectively. This dual role leads to a cooperative effect that enhances the efficiency of HER. As an example of a monometallic TMP electrocatalyst, Shen et al. prepared cobalt phosphide (CoP_x_) nanoparticles that showed the overpotential of 184 at 10 mA cm^−2^ for the HER in 1 M KOH, an overall water splitting performance with 10 mA cm^−2^ at 1.58 V, and a long-time reaction stability without attenuation during 10 h operation [16].

Substantial progress has been achieved in further promoting the electrocatalytic activity and stability of the TMPs through different design strategies. One of these strategies has focused on avoiding the agglomeration of the TMP particles by using phytic acid (PA) as a P source. With the strong chelating capacity and high phosphorus content, PA forms a complex with the metal ions and disperses them, obtaining homogeneously distributed TMP nanoparticles after calcination. Moreover, different from other P sources, PA is also a nontoxic, natural, and environmentally friendly biogenic organic phosphorus source. According to this, Pu et al. synthesized different types of TMP nanoparticles (FeP, CoP, and Ni_2_P) encapsulated in N and P co-doped carbon using phytic acid and melamine as a source of P and carbon, respectively [17]. The phytic acids formed a crosslinked self-assemble complex with respective metal ions with good results.

Other common strategies to improve TMP electrocatalytic activity and stability are based on their attachment to a proper substrate to avoid particle agglomeration but also improve the poor electrical conductivity and stability of TMPs. Carbonaceous materials modified with the TMPs for overall water splitting have demonstrated to be a type of functional material with exceptional stability, conductivity, modulated porosity, and unique three-dimensional architectures. Conductive carbon-based nanomaterials, such as graphene, carbon nanosheets, carbon nanorods, carbon nanotubes, and carbon nanofibers (CNFs), have been explored as excellent electrode support materials [18,19,20]. From all of them, the CNFs with adjustable composition, large length–diameter ratio (>1000), high surface area, and three-dimensional network structure provide excellent electrical conductivity and promising electrochemical stability. Additionally, natural defects and functional groups of nitrogen-doped carbon nanofiber contribute to increasing active sites of the electrocatalyst [21]. The electrospinning technique to fabricate CNFs is a process that provides versatility to the electrocatalytic system regarding: (a) homogeneous incorporation of the catalytic materials into the carbon structure; (b) high catalyst-specific surface area easily accessible to reactants; (c) high stability of the nanoparticles with low agglomeration and leaching of the catalysts during the reaction; (d) high electronic conductivity of the nanocomposite; and (e) it is an up-scalable process [22]. Examples of TMP-supported CNFs include the work of Ren et al. [23] that prepared CoP-supported CNFs by an electrospinning process followed by a thermal treatment and a phosphorization step with sodium hypophosphite (NaH_2_PO_2_). This CoP-CNF electrocatalyst showed a low overpotential of 127 mV at 10 mA cm^−2^ for the HER in 1 M KOH. In another study, Wang et al. [24] demonstrate that the resistivity of Ni_2_P was greatly decreased (10^4^) when it was incorporated in CNFs, providing values of HER overpotential of 185.3 and 104.2 mV in neutral and basic media, respectively. The well-dispersed Ni_2_P nanoparticles through the microporous CNFs with high surface areas are the main reasons for this enhanced electrocatalytic behavior.

In the previous mentioned studies, almost all the cathodes have been prepared by mixing a controlled quantity of the synthesized electrocatalysts (corresponding to the mass loading) with a binder (generally an ionomer; Nafion) and a solvent and, finally, applying and drying the ink onto a gas diffusion layer (GDL). However, a more efficient way is to directly use the free-standing films of these TMP-supported CNFs as cathodes, with no need to prepare, apply, and dry any ink onto a GDL. According to this, several studies have been identified in the literature using TMP-supported CNF films as free-standing electrodes for the HER. Regarding the monometallic TMP, Lu et al. [25] prepared self-supporting CoP-CNF composite membranes with lotus root-like structure showing high performance for the HER in all pH ranges. This is explained due to the CNFs longitudinal channels integrated with mesopores and their conductivity, which efficiently improve the contact between CoP and the electrolyte and forms an interconnected conductive network. In another study, Quan et al. [26] prepared various TMP-supported CNF composite films by electrospinning and using phytic acid as a P source. The best electrodes (Co_2_P-CNF films) showed an overpotential of 275 mV at 10 mA cm^−2^ in 1 M KOH. It is demonstrated that the carbon-based fibrous structures offer faster charge transfer pathways and an enlarged active surface area than the prepared carbon-based particles.

In this work, we have successfully developed free-standing CNF films with supported cobalt phosphide nanoparticles through an electrospinning process and a thermal treatment under controlled conditions. We used phytic acid as a phosphorus source during the electrospinning process, which enabled the homogeneous incorporation of cobalt phosphide nanoparticles onto the CNFs. This synthesis approach led to films with exceptional physicochemical properties, including a high surface area, porosity, catalytic mass loading, and electrical conductivity, and with enough stability to be used directly as cathodes. Electrochemical characterization in alkaline media revealed promising HER performance. Moreover, these free-standing cobalt phosphide-supported CNF films exhibited excellent performance and stability when assessed as cathodes in a MEC reactor, facilitating H_2_ production at a remarkably low operational cell voltage. This innovative material demonstrates significant potential for efficient and sustainable H_2_ generation in alkaline systems.

## 2. Materials and Methods

### 2.1. Materials for Synthesis

Polyacrylonitrile (PAN, Sigma-Aldrich, St. Louis, MO, USA), N,N-dimethylformamide (DMF, Scharlab, Barcelona, Spain), phytic acid solution (50% (*w*/*w*) in H_2_O, Sigma-Aldrich), and cobalt nitrate hexahydrate (Co(NO_3_)_2_·6H_2_O, Sigma-Aldrich) were used for the synthesis.

### 2.2. Synthesis of Free-Standing Cobalt Phosphide-Supported CNF Films

The preparation of the cobalt phosphide-supported CNF films was performed by an electrospinning process followed by a thermal treatment. For the electrospinning process, a precursor polymeric solution was prepared by dissolving 8 g of PAN in 52 g of DMF by mechanical stirring during 12 h at 60 °C. Phytic acid was used as the P source, and since the commercially available phytic acid contained a high H_2_O amount (50 wt.% H_2_O), first, an evaporation step to eliminate all the water using the rotavapor was performed. Then 2.56 g of the phytic acid and 1.6 g of Co(NO_3_)_2_·6H_2_O were dissolved in 16 g of DMF by mechanical stirring during 12 h at room temperature. After, both solutions were mixed before the start of the electrospinning process to obtain a homogenous mixture. For the electrospinning process (MECC Co., Ltd., Fukuoka, Japan, model NF-103), the following conditions were used: flow rate of 2.2–2.5 mL h^−1^, voltage of 29 kV, collector cylinder speed of 300 rpm, needle to collector distance of 14.5 cm, three syringes with 21-stainless steel needles, temperature approx. 21 °C, and relative humidity approx. 45%. The process took more or less time to obtain different sample thicknesses (approx. from 6 to 14 h, this last one with higher thickness values). The resulting nanofiber films were thermally treated in two steps: an oxidizing step at 280 °C for 1.5 h in air with a heating rate of 1 °C min^−1^, to stabilize the PAN fibers, followed by a carbonization step at 1000 °C for 1 or 2 h in an argon atmosphere with a heating rate of 5 °C min^−1^, to obtain the CNF films. Samples were named Co_2_P@1h(160) for the sample treated 1 h at 1000 °C, and Co_2_P@2h(120), Co_2_P@2h(140), and Co_2_P@2h(165) for the samples treated for 2 h at that temperature. Differences between them are related to the CNF film thickness, in µm, which is indicated between brackets. For the test in the MEC reactor, a CNF film of 12 × 12 cm^2^ was used (sample Co_2_P@2h(140)).

The oxidized CNFs were fabricated according to the methodology described in the previous part, using the sample Co_2_P@2h(140), followed by a second thermal treatment at 300 °C for 0.5 h in air, with a heating rate of 1 °C min^−1^, to partially oxidize the Co. The product is named Co_2_Pox@2h(110).

For comparison reasons, pristine CNF films have also been fabricated following the same procedure described above but without using the Co and P precursors. For this, 8 g of PAN was dissolved in 72 g of DMF by mechanical stirring during 12 h at 60 °C. Then, the electrospinning process was performed using three 20 mL syringes with 21-stainless steel needles, a high voltage (29.0 kV) at 2.5–2.7 mL h^−1^ flow rate, a needle to collector distance of 15 cm, and a collector cylinder speed of 300 rpm, with a relative humidity of 45% and a temperature of about 21–23 °C. Finally, an identical two-step thermal treatment was followed to achieve the pristine CNF films: CNF@2h(140).

### 2.3. Physicochemical Characterization

Brunauer–Emmett–Teller (BET) measurements were performed in a surface area and pore volume analyzer (NOVA2200e, Quantachrome Instruments, Boynton Beach, FL, USA) using sample strips placed in the bulb cells and by using N_2_ as the absorbent gas to determine the specific surface area, total pore volume, and percentage of microporosity vs. mesoporosity. An inductively coupled plasma mass spectroscopy (ICP-MS) analysis was performed on an ICPMS Agilent Serie 7500 (Agilent, Santa Clara, CA, USA). Before its measurement, the samples were weighted, digested in an acid solution (HNO_3_ 70%), and placed on an analytical microwave at 250 °C. Then, the digestion residue was diluted to analyze the desired elements. The conductivity analysis was carried out with homemade equipment using the multimeter Lexman PT1000 (Lexman, Alfortville, France). For this, a support made of Teflon with 4 copper strips was used, in which a CNF film area of 2 × 2 cm^2^ was allocated. The measured resistance was transformed into S m^−1^ with the thickness measurements. X-ray diffraction (XRD) analysis was performed from 20 to 60 degrees on a diffractometer Malvern PANalytical X’pert Pro MPD (XRD Facility, Institut Català de Nanociència i Nanotecnologia) at room temperature. X-ray photoelectron spectroscopy (XPS) analysis was performed in a SPECS system with a PHOIBOS 150 EP hemispherical energy analyzer with an MCD-9 detector (XPS Facility, Universitat Politècnica de Catalunya, Barcelona, Spain). Raman spectra were collected in a dispersive spectrometer, Jobin-Yvon LabRam HR 800 (Horiba, Kyoto, Japan), coupled to an optical microscope, Olympus BXFM (Olympus, Tokyo, Japan), and a 532 nm diode-pumped solid-state laser was used as an excitation source (Universitat de Barcelona, Barcelona, Spain). Scanning Electron Microscopy (SEM) analysis was carried out in a Zeiss Merlin FE-SEM (Carl Zeiss Microscopy, Universitat Autònoma de Barcelona, Barcelona, Spain) without metallization of samples. Images were recorded at different steps of magnification. Transmission Electron Microscopy (TEM) analysis was performed on a JEOL JEM-2011 TEM (Universitat Autònoma de Barcelona, Barcelona, Spain) using an acceleration voltage of 200 kV in bright field and in diffraction mode.

### 2.4. Electrochemical Characterization

The CNF film electrodes have been electrochemically characterized using the BioLogic VMP3 potentiostat/galvanostat (BioLogic, Grenoble, France). A three-electrode electrochemical cell (EL-ELECTRO-150DJ from BioLogic) has been employed, consisting of a reversible H_2_ electrode (HydroFlex RHE) as reference electrode (REF), a Pt wire as counter electrode (CE), and the working electrode (WE) made from a stainless-steel mesh isolated with a film of plastic. A circular portion (0.126 cm^2^ area) of the material to be studied is placed inside the stainless-steel mesh exposed to 1 M KOH, which is used as electrolyte (80 mL).

Electrocatalytic performance of different developed CNFs was evaluated for HER. This characterization was carried out by applying cyclic voltammetry (CV) and linear sweep voltammetry (LSV). HER was performed with the following steps: (1) bubbling *N*_2_ (25 min), (2) OCV (5 min), (3) CV (5 cycles), 25 mV s^−1^ scan rate, from 0.10 to −0.60 V (vs. RHE), (4) OCV (5 min), (5) LSV, 1 mV s^−1^ scan rate, from 0.10 to −0.70 V (vs. RHE), and (6) OCV (5 min).

To assess the performance of the CNFs in an electrolysis cell, the upscaled sample Co_2_P@2h(140) was also tested in a double-chamber flat plate MEC reactor. A cationic exchange membrane (RALEX^®^ CMHPP, from MEGA, Prague, Czech Republic) was placed in the middle, sandwiched between the anode and cathode. The anode was a carbon felt (Sigracell KFD 2.5 EA, from SGL Carbon, Wiesbaden, Germany), which supports the biofilm growth, acting as a bioanode. The cathode used as a reference material was a carbon cloth with platinum on carbon catalyst (0.5 mg cm^−2^ 60% wt.% Pt on Vulcan cloth electrode, from FuelCellStore, Bryan, TX, USA). The cobalt phosphide-supported CNF films were used directly as cathodes. Anode and cathode had a current collector made of stainless steel 316L. The active area of the anode, membrane, and cathode was 100 cm^2^. The volume of anode was 200 mL with a buffer tank of 1 L integrated in the recirculating loop. The volume of the cathode was 200 mL. The anolyte composition was: NaHCO_3_ (1.5 g L^−1^), K_2_HPO_4_ (0.3 g L^−1^), MgSO_4_ (0.1 g L^−1^), CaCl_2_ (0.05 g L^−1^), NH_4_HCO_3_ (14.37 g L^−1^), and NH_4_CH_3_COO (2.5 g L^−1^) and the catholyte was the same without NH_4_HCO_3_, nor NH_4_CH_3_COO. The MEC reactor was operated at 30 °C and ambient pressure in a two-electrode configuration, applying a cell voltage of 0.75 V for more than 130 h. At this point, polarization curves (*j* vs. *V*), applying voltages in the range of 0.25–1.25 V, and constant voltage techniques were conducted to assess the performance of both cathode materials working at the MEC reactor.

### 2.5. Calculations

The cathode efficiency of MEC reactor is evaluated assuming there is only one reduction reaction (no other secondary reactions that consume the energy demanded by the system are observed), which is the HER, and that H_2_ behaves like an ideal gas, so the theoretical H_2_ produced is given by the following formula:

(1) $$H_{2}\mathrm{theor}.=\frac{I\left(A\right)\cdot t(s)}{F\cdot e^{-}\cdot R\cdot T(K)}$$

where *I* is the current intensity, *t* is the experiment duration, *F* is the Faraday constant, *e^−^* electrons involved in the reaction (two), *R* is the ideal gas constant, and *T* is the temperature.

The energy efficiency has been evaluated considering just the energy input demanded by the reactor itself, which means the contribution of all the peripherals, such as peristaltic pumps and external heating systems, is not included at this point. This efficiency has been expressed as the specific energy consumption, SEC, required for theoretically H_2_ produced:

(2) $$\mathrm{SEC}(\mathrm{kWh}{\mathrm{kg}}^{-1}H_{2}\mathrm{theor}.)=\frac{I\left(A\right)\cdot V\left(V\right)\cdot t(s)}{m\;H_{2}\;theor.\left(kg\right)\cdot 1000}$$

where *V* is the applied voltage and H_2_ theor. mass can be easily deduced from the integral of electrical current demand.

Finally, the H_2_ production rate is determined by the following equation:

(3) $$\mathrm{Theoretical}\;H_{2}\;\mathrm{prod}.\;\mathrm{rate}(L\;H_{2}\;m^{-3}\mathrm{reactor}\;\mathrm{and}\;{\mathrm{day}}^{-1})=\frac{H_{2}\;theor.}{V_{reactor}\;(m^{3})\cdot t\;(d)}$$

## 3. Results and Discussion

### 3.1. Synthesis

**Figure 1:**
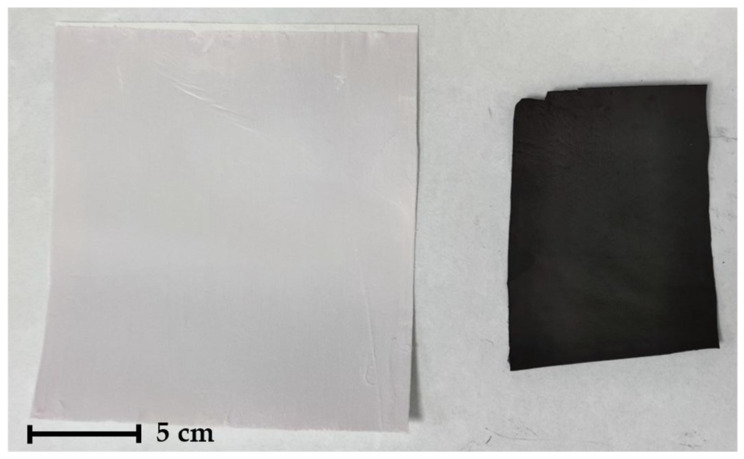
Pictures of the nanofiber films before (**left**) and after (**right**) the thermal treatment.

The different CNF-based films were prepared by an electrospinning process followed by a thermal treatment. In the first step, the precursor solution for the electrospinning process was prepared using PA as the P source with the cobalt nitrate as the TM source. The use of PA with six phosphate groups can easily chelate with the Co ions to form a complex, which is good for the dispersion of the metal ions and to obtain homogeneously dispersed cobalt phosphide nanoparticles through the CNFs after the thermal treatment. After the electrospinning process, a mat of PAN nanofibers with supported Co nitrate and Co-PA is obtained. During the thermal treatment, a first stabilization step takes place under air in which the PAN chains are cycled to promote their cross-linking and the thermally stable ladder-like structure, which avoids fusing during the next carbonization process. Through the carbonization phase, the P-O bonds in the Co-PA are broken at high temperature by the carbon reduction under an argon atmosphere, and the released P is doped into the lattice of the Co to eventually form cobalt phosphide nanoparticles. Figure 1 shows the nanofiber films before and after the thermal treatment. An important shrinkage around 30% in the x and y direction takes place during the thermal treatment that is due to the different reactions occurring, giving an important weight loss because of the emission of different outgases (CO, H_2_O, N_2_, HCN, etc.). Therefore, an approximate yield value after the thermal treatment is in the range between 30 and 40%.

### 3.2. Physicochemical Characterizations

Different physicochemical characterizations were carried out to evaluate the properties that play an important role in their end-use as an electroactive catalyst.

The nitrogen adsorption and desorption analysis confirm the high surface area (S.A.) of all the synthesized CNFs (up to 502 m^2^ g^−1^ by BET method) with total pore volume values (V) up to 0.33 cm^3^ g^−1^, as it is visualized in Table 1. Values of surface area (S.A.), pore volume (V), thickness, and ICP-MS of all samples. The t-plot method determines that this pore volume is due to the presence of both micropores and mesopores equally distributed (approx. 50% of each). In the same way, the pristine CNFs show similar surface area and pore volume values. The Co_2_P@2h samples showed higher porosity than the Co_2_P@1h sample, and the Co_2_Pox@2h shows the highest porosity value. The additional thermal treatment and under oxygen can explain these results.

Measured thickness values were in the range of 110 to 165 µm (Table 1). This value depends on the duration of the electrospinning process, being higher with a longer time of the electrospinning process, and on the thermal treatment, being higher with a shorter thermal treatment. It is included in the table due to the important influence on the electrochemical performance, as it will be commented below. It is important to consider that these values are variable within a film and, therefore, the value is the average from several measured ones.

ICP-MS analysis reveals contents of Co and P in the ranges of 8.6–12 and 2.6–4.7 wt.%, respectively. In general, Co and P content increases with film thickness and with thermal treatment time. Therefore, the sample Co_2_P@2h(165) shows the highest Co and P content, and the samples Co_2_P@1h(160) and Co_2_P@2h(120) have the lower content. According to this analysis, a mixture of CoP and Co_2_P is present in the samples. However, to better determine which is the present crystalline phase of the samples, XRD analysis was performed.

The in-plane electrical conductivity of the films has also been measured, and the values are in the range of 280–2000 S m^−1^. Noteworthy that these values are obtained with an in-house-made equipment and using the average thickness values. Both factors can induce high variability in the measured values. As a general trend, the use of the CNFs with supported cobalt phosphide increases the electrical conductivity in comparison with pristine CNFs. Moreover, higher thermal treatments and higher thicknesses also increase the conductivity. On the contrary, the oxidized sample presents a lower conductivity.

**Figure 2:**
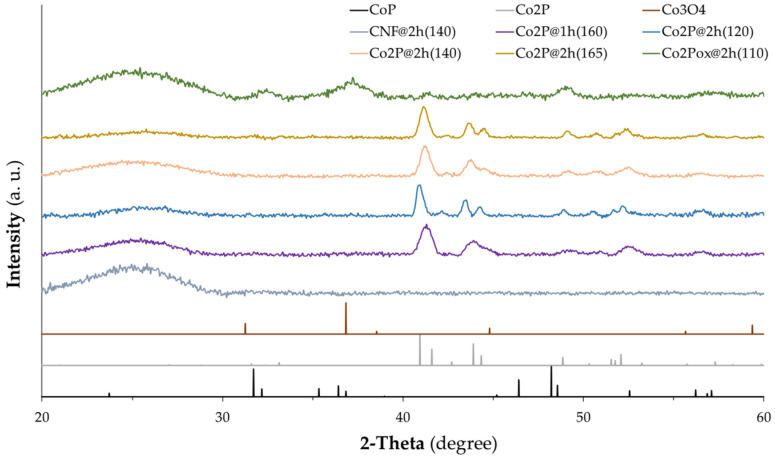
XRD patterns of all CNFs samples including CoP, Co_2_P, and Co_3_O_4_ diffraction patterns.

To confirm the crystalline phase structure of the electrocatalysts in the CNFs, XRD analysis was employed, and the results are shown in Figure 2. For all the samples except the oxidized one, it is observed that the most intense peak appears at 41°, which corresponds to the lattice plane (121) of the Co_2_P orthorhombic phase (JCPDS No. 32-0306). Moreover, the two peaks found around 44° and the ones at 48° and 52° are also related to that Co_2_P crystal phase. This confirms the presence of Co_2_P nanoparticles in the CNFs. In the case of the oxidized sample, additionally to the presence of Co_2_P, the peak at 32° revels the presence of CoP (JCPDS No. 29-0497) and the peak at 37° of Co_3_O_4_ (JCPDS No. 42-1467). In all samples, the broad peak near 25° corresponds to the graphitic carbon plane (002). In all samples, except the oxidized one, the CoP crystalline phase cannot be detected, which is not in agreement with the ICP-MS analysis. The error in the ICP-MS analysis (up to 3%) and the possible presence of amorphous CoP could be possible explanations for this.

**Figure 3:**
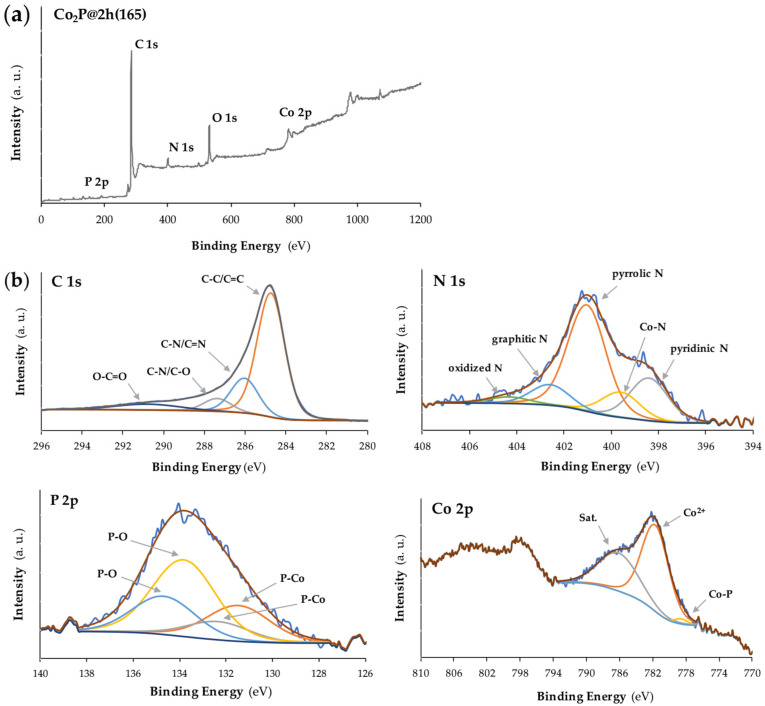
XPS of Co_2_P@2h(165): (**a**) full spectrum, and (**b**) high resolution XPS spectra for C 1s, N 1s, P 2p, and Co 2p, respectively.

XPS analysis was employed to assess the chemical oxidation state on the surface of the best-performing sample, Co_2_P@2h(165). The presence of C, N, Co, and P elements is shown in Figure 3. For the C 1s spectra, the four peaks at 285.0 eV, 286.0 eV, 287.0 eV, and 290.0 eV indicated the presence of C-C/C=C, C-N/C=N, C-N/C-O, and O-C=O bonds, respectively [27]. The C-N bond in the C 1s XPS spectrum confirmed the N doping in the samples. The N 1s spectrum shows the presence of five peaks at 398.0 eV, 399.0 eV, 401.0 eV, 403.0 eV, and 404.0 eV, indicating pyridinic, Co-N bond, pyrrolic, graphitic, and oxidized N, respectively [28]. Due to the electronegativity difference between nitrogen and carbon, the N-doping can induce adjacent positively charged carbon atoms, increasing the electrical conductivity and the electrocatalytic activity [29]. For the Co 2p and according to the literature, the peak located at 778.5 eV (2p_3/2_) is assigned to the presence of the partially positive charged Co-P bond in Co_2_P, which shifts from the metallic Co (777.9 eV). The peak at 782.0 eV (2p_3/2_) indicated the surface oxidized Co, and a satellite peak can be found at 786.5 eV [25,26,28,29]. For the P 2p spectrum, the two peaks at 131.5 eV (2p_1/2_) and 132.5 eV (2p_3/2_) can be assigned to P in metal phosphide and the two at 134.0 eV and 135.0 eV can be assigned to P-O from the phosphate, because of the partial oxidation of phosphide in air [26,29].

**Figure 4:**
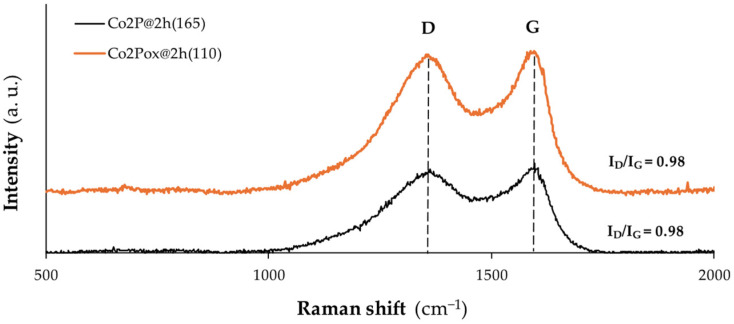
Raman spectra of the samples Co_2_P@2h(165) and Co_2_Pox@2h(110).

The verification of carbon structure was performed by Raman spectroscopy (Figure 4) for the samples Co_2_P@2h(165) and Co_2_Pox@2h(110). The Raman spectroscopy determines the presence of graphite and defects, which are supposed to increase electrical conductivity and chemical reactivity to the cathode material, respectively. Mainly two peaks could be identified: the D band (1350–1370 cm^−1^) and the G band (1580–1600 cm^−1^), corresponding to disordered carbon (defects) and crystalline graphite, respectively. The degree of defect can be represented by the intensity ratio of two peaks (I_D_/I_G_). In both samples, the ratio I_D_/I_G_ was 0.98, which indicates a representative graphitization degree, considering that all the nanofibers were pyrolyzed at 1000 °C with no change after the oxidation step.

**Figure 5:**
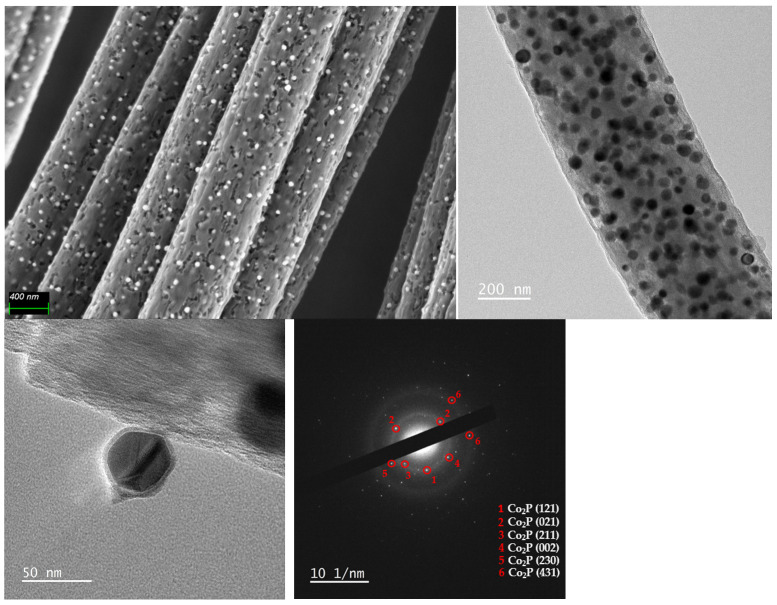
HRSEM and TEM images of the sample Co_2_P@2h(165) (**above**) and SAED pattern (**below**).

The morphology of the sample Co_2_P@2h(165) was visualized by HRSEM and TEM analysis (Figure 5). Both images show homogeneously distributed cobalt phosphide nanoparticles (less than 100 nm) embedded inside and outside the CNF matrix. The electron diffraction pattern taken from selected area electron diffraction (SAED) (Figure 5 below) confirmed the presence of crystalline Co_2_P nanoparticles with the diffraction rings corresponding to the planes (121), (021), (211), (002), (230), and (431).

### 3.3. Electrochemical Characterizations

**Figure 6:**
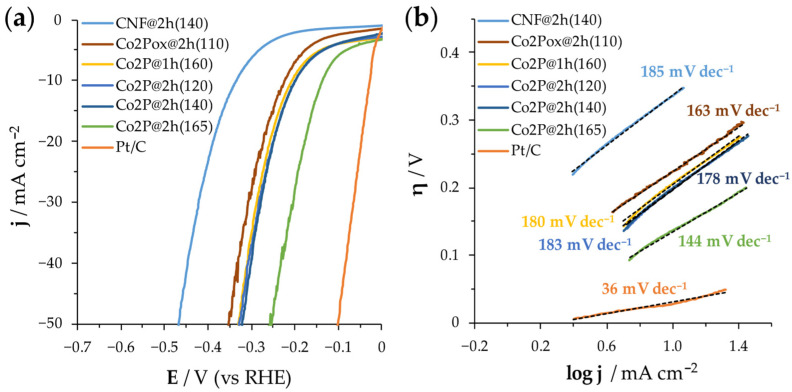
LSV curves (**a**) and Tafel plots (**b**) for CNFs samples compared to the Pt/C used as reference.

H_2_ evolution LSV curves and Tafel plots were measured for all the CNFs developed and compared to commercial Pt/C samples (Figure 6 and Table 2).

The best results were found for Co_2_P@2h(165), (*η*_10_ = 137 mV), as expected, due to the higher mass loading of catalyst (0.49 mg cm^−2^, calculated respect the Co content), associated with its larger thickness (165 μm, see Table 1). It is noticeable that Co_2_P@2h(165) behaves better not only at lower current densities but also at higher values, increasing the overpotential 46% when current density is five times higher (*η*_50_ = 255 mV) and 61% when it is ten times higher (*η*_100_ = 354 mV), which is lower than the increase observed for Pt/C (72% and 84% for *η*_50_ and *η*_100_, respectively). That better performance compared, for instance, to Co_2_P@1h(160), which shows a similar thickness, could be attributed to both higher porosity and conductivity found after the two hours of thermal treatment. Moreover, the morphology of the sample with a 1D porous carbon structure not only increases the active surface but also the charge transfer through the carbon backbone and the contact between the catalyst and electrolyte. The uniformly distributed small cobalt phosphide nanoparticles result in a large contact area between the electrolyte and catalyst, and the contact between carbon and cobalt phosphide also reduces the interfacial resistance. However, the Tafel slopes shown in Figure 6b point out that Pt/C still will be achieving very low overpotentials when it comes to much higher values of current densities, with a slope of only 36 mV dec^−1^ compared to the 144 mV dec^−1^ exhibited by the Co_2_P@2h(165) sample. The pristine CNF, CNF@2h(140), presents very high overpotentials and Tafel slope values, indicating poor activity and the relevance of the cobalt phosphide nanoparticles as HER catalysts. It is outstanding that the performance keeps almost the same between the sample Co_2_P@2h(120) (*η*_10_ = 198 mV) and the sample Co_2_P@2h(140) (*η*_10_ = 199), which emphasizes that the scalability process has not affected the electrochemical properties. Sample Co_2_P@1h(160), although it has less surface area, shows similar overpotentials to those of the Co_2_P@2h(120) and Co_2_P@2h(140) samples. The higher thickness and higher mass loading of these samples (160 µm) can explain this result. Finally, the oxidated sample Co_2_Pox@2h(110) shows the poorest HER performance among all the supported samples, suggesting both that oxidized cobalt species and thinner materials do not have a beneficial effect, despite the significant increase in surface area.

Table 3 summarizes the literature findings for some similar TMP-supported carbon-based materials.

These materials present a similar mass loading to the Co_2_P@2h(165) sample, ranging between 0.28 and 1.00 mg cm^−2^. The highest HER performance is observed with Ni_2_P@NPCNF, with an overpotential of 104 mV. CoP-based CNF materials display comparable overpotentials at *η*_10_ to Co_2_P@2h(165), though they demonstrate lower Tafel slopes. Note that materials tested in acidic electrolytes generally exhibit better HER performances than those tested in alkaline conditions, due to the higher free energy needed for the alkaline reaction [14,25,26,36,37]. Thus, the Co_2_P@2h(165) developed in this study shows a high performance compared to the reported materials in the literature. However, most of these materials are typically synthesized as powders, requiring additional steps to fabricate electrodes. In this regard, Co_2_P@2h(165) presents a distinct advantage, as it is a free-standing carbon material that can be used directly as an electrode without further processing.

### 3.4. Electrochemical Stability Evaluation

**Figure 7:**
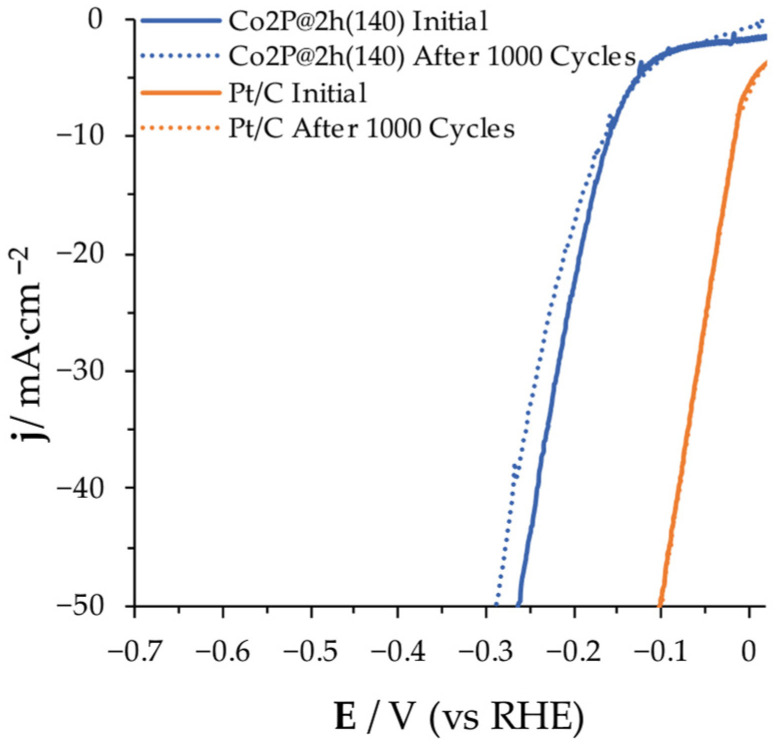
LSV performed at initial (straight lines) and after 1000 cycles (dotted lines) for Pt/C and Co_2_P@2h(140) electrodes.

Cyclic voltammetry in 1 M KOH solution was conducted for 1000 cycles at 25 mV s^−1^ of scan rate between 0.10 and −0.60 V (vs. RHE) for assessing the long-term steadiness. Figure 7 shows the performance of the Co_2_P@2h(140) that was used as the cathode in the following MEC reactor tests, compared to that found for the Pt/C electrode used as a reference.

It can be observed that the Pt/C electrode shows almost no loss across the entire range of current densities assessed after the 1000 cycles. In relation to Co_2_P@2h(140), the overpotential at lower current densities (until approximately 10 mA cm^−2^) remains nearly constant, with a slight increase at higher current densities. Although the performance of Co_2_P@2h(140) is significantly below that of the Pt/C electrode, in terms of stability, it highlights when compared to other TMPs of similar chemical structures reported in the literature [13]. The use of CNFs as supporting material provides enhanced electrochemical stability to the cobalt phosphide nanoparticles, avoiding their agglomeration with no need of a binder.

### 3.5. In Situ Bio-Electrochemical Reactor Testing

**Figure 8:**
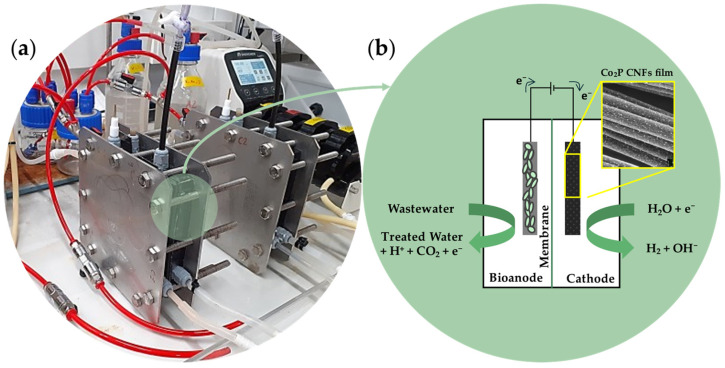
MEC reactors for assessing cathodes performances (**a**) and scheme of functioning (**b**).

The Co_2_P@2h(140) sample that was synthesized at a larger scale was tested directly as a cathode in a MEC reactor, using an area up to 12 × 12 cm^2^. It is important to remark that the cobalt phosphide-supported CNF films can be used directly as electrode materials without the need to obtain a powder and use binders and solvents, which greatly simplifies the electrode preparation process. The MEC reactor (Figure 8) was also operated with Pt/C-based cathode as high-performance reference material.

**Figure 9:**
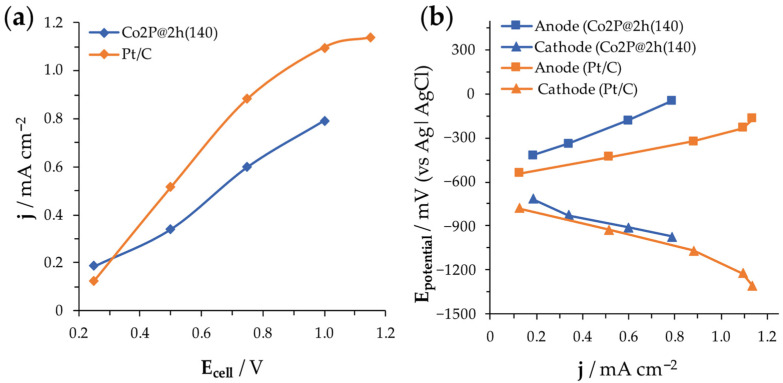
Polarization curves, j vs. V, (**a**) and electrode potentials followed up (**b**) for comparison of Co_2_P@2h(140) and Pt/C cathodes while operating in MEC reactors.

The polarization curves shown in Figure 9a indicate that Co_2_P@2h(140) offers a competitive performance in comparison to reference material (Pt/C cathode). The MEC reactor operating with Pt-based cathode achieves 1.1 mA cm^−2^ (11 A m^−2^), which is a high current density. The MEC reactor using Co_2_P@2h(140) cathode achieves a current density of 0.8 mA cm^−2^ (8 A m^−2^), representing a 25% decrease compared to that for Pt-based cathode. Notably, the Co_2_P@2h(140) is a free-PGM material, making its performance particularly significant. The current densities reported in the literature generally range from 1 to 15 A m^−2^, with the highest values reported by Rossi et al. being 43 A m^−2^, using Pt/C, and 48 A m^−2^ with NiMo cathodes, both measured in very small MEC reactors where the total volume is just 4.5 mL [38,39,40,41]. Figure 9b depicts the three-electrode polarization curves, where anode and cathode performances are evaluated through the evolution of their overpotentials. At higher current densities in the polarization curves (corresponding to higher applied voltages), the potentials of the anodes increase towards more positive values. The anode potential increases because it needs to transfer electrons at a faster rate, and this creates the overpotential. Notably, the anode potentials in both configurations did not increase to positive potentials, preventing biofilm degradation [42]. The anode of the MEC reactor operated with Co_2_P@2h(140) exhibits a faster increase in anodic overpotential. To avoid potential anode degradation, higher current densities were not explored in the reactor with this material. The overpotentials of the cathodes show a very similar trend across the studied current density range (0.1 to 0.8 mA cm^−2^). This observation suggests comparable performance between the Co_2_P@2h(140) cathode and the Pt-based reference cathode within the mentioned current density range. The specific structure of the cobalt phosphide nanoparticles supported on CNFs with a homogeneous distribution, high surface area, porosity, and electrical conductivity allows a high active surface area, high contact between the electrode, and electrolyte and high charge transfer among the CNF matrix. The intimate contact between the catalysts and electrode leads to a shorter OH^−^ diffusion pathway and a low interfacial resistance. Moreover, the direct use of the film as a cathode without the need to use a binder increases the active sites and the mass/electron transport. All these physicochemical properties contribute to the good electrochemical performance.

The Co_2_P@2h(140)-based cathode has been operated in a MEC reactor undergoing three consecutive cycles of 45 h each, for a total duration of 135 h. Considering the results from the polarization curve (Figure 9b), the voltage applied was 0.75 V (as well as for the Pt/C cathode), and the current density was consistent throughout the entire process. H_2_ production was calculated based on the current density demand, assuming a Faradaic efficiency of 100%. MEC operating with Pt/C cathode demonstrates superior performance, achieving an average current density of 9.1 A m^−2^ compared to 5.4 A m^−2^ for the MEC with Co_2_P@2h(140) cathode. These current densities corresponded to total theoretical H_2_ productions of 4.62 L vs. 2.84 L, respectively, translating to H_2_ production rates of 2410 m^3^ H_2_ m^−3^ reactor and day^−1^ and 1482 m^3^ H_2_ m^−3^ reactor and day^−1^, respectively. Although the productivities were initially calculated assuming a 100% Faradaic efficiency, experimental analysis of the collected gas revealed a Faradaic efficiency of 67% for the Pt/C cathode. This reduction was primarily attributed to gas leakages during reactor operation and gas analysis, since cathodic parasitic reduction reactions were not observed.

In terms of energy efficiency, Pt/C also outperforms Co_2_P@2h(140) with a lower theoretical specific energy consumption (SEC) of 20.1 kWh kg^−1^ H_2_ (30.3 kWh kg^−1^ H_2_ considering the experimental quantified H_2_ production) compared to 26.8 kWh kg^−1^ H_2_ for Co_2_P@2h(140). Considering the lower heating value of H_2_, which is 33.3 kWh kg^−1^ H_2_, the energy efficiencies for MEC using both cathodes exceed 100%, markedly higher than the typical efficiencies of 55−65% reported in conventional water electrolysers. In MECs, H_2_ production is not coming from the reverse combustion reaction of H_2_, thus the process requires less electrical energy than water electrolysis, leading to energy yields over 100%. This phenomenon arises from the synergistic contribution of energetic power and chemical energy derived from oxidized compounds at the bioanode [8,43]. While Pt/C cathode demonstrates a lower energy requirement for H_2_ production, thus positioning it as the most efficient material overall, its high cost associated with Pt content may restrict its applicability for large-scale implementation. The Co_2_P@2h(140) cathode, despite being less efficient, represents a cost-effective alternative for scenarios where budget constraints are a concern. Depending on the priorities of the MEC system, either material could be a viable option, aiming to achieve an optimal balance between efficiency and cost.

**Figure 10:**
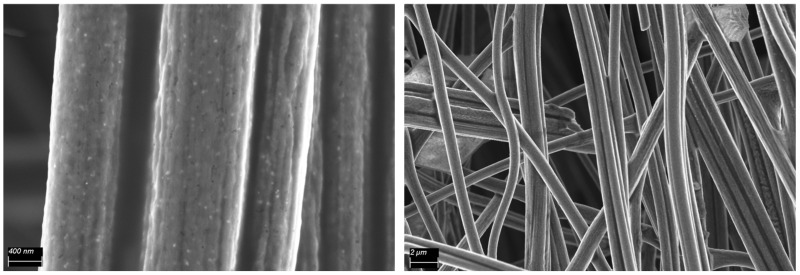
HRSEM images of Co_2_P@2h(140), after being operated in the MEC reactor.

In Figure 10, the HRSEM post-mortem images of sample Co_2_P@2h(140) used in the MEC reactor show that both CNFs and cobalt phosphide nanoparticles did not change after the electrochemical process. Even though some agglomerations of possible salts from the electrolyte were visualized between the CNFs. This analysis confirms the stability of the cathode.

## 4. Conclusions

In summary, the cobalt phosphide-supported carbon nanofiber films developed in this study offer a significant advantage over most electrodes used for the HER, which are typically synthesized as powders and require additional steps for electrode fabrication. These cobalt phosphide CNF films can also be utilized directly as electrodes without further processing, thanks to their favorable characteristics, including high surface area, porosity, electrical conductivity, and catalyst mass loading. The electrochemical characterizations revealed an overpotential lower than 140 mV at 10 mA cm^−2^ for the HER in alkaline media (1 M KOH), and the stability test indicated outstanding steadiness. Additionally, they showed a promising application as cathodes in a MEC reactor, exhibiting a current density demand of 5.4 A m^−2^ with low cell voltage (0.75 V) applied. It is important to emphasize that since the electrospinning is an up-scalable process, these free-standing CNF films have the potential to be produced at a larger scale. The next studies will be focused on bimetallic phosphide-supported CNF films, which could further improve the performance of monometallic phosphides prepared in this work, and they will be tested as cathodes in AEM electrolysers.

## Figures and Tables

**Table 1:** Values of surface area (S.A.), pore volume (V), thickness, and ICP-MS of all samples.

Sample	S.A. (m^2^ g^−1^)	V (cm^3^ g^−1^)	Thickness (µm)	ICP-MS (wt.%)
CNF@2h(140)	431	0.27	140	0
Co_2_P@1h(160)	269	0.23	160	8.7 Co, 2.6 P
Co_2_P@2h(120)	358	0.31	120	8.6 Co, 3.0 P
Co_2_P@2h(140)	390	0.29	140	9.7 Co, 4.7 P
Co_2_P@2h(165)	411	0.33	165	12.0 Co, 4.2 P
Co_2_Pox@2h(110)	502	0.32	110	9.9 Co, 4.3 P

**Table 2:** Comparative of HER overpotentials (*η*) and Tafel slopes of the CNFs and Pt/C.

Sample	Overpotentials (mV)			Tafel Slope (mV dec^−1^)	Catalyst Mass Loading (mg cm^−2^)
	*η*_10_	*η*_50_	*η*_100_		
Pt/C	29	103	185	36	0.50
CNF@2h(140)	335	468	547	185	0
Co_2_P@1h(160)	207	329	406	180	0.39
Co_2_P@2h(120)	198	328	413	183	0.24
Co_2_P@2h(140)	199	323	395	178	0.29
Co_2_P@2h(165)	137	255	354	144	0.49
Co_2_Pox@2h(110)	224	353	426	163	0.37

**Table 3:** HER performance comparison among different TMP derivatives reported in the literature and the best materials assessed in this study.

Sample	Electrolyte	Overpotential, *η*_10_ (mg cm^−2^)	Tafel Slope (mV dec^−1^)	Catalyst Mass Loading (mg cm^−2^)	Reference
Pt/C	1 M KOH	29	36	0.50	This work
Co_2_P@2h(165)	1 M KOH	137	144	0.49	This work
CoP@CNF	1 M KOH	127	73	0.51	[23]
Ni_2_P@NPCNF ^1^	1 M KOH	104	80	0.34	[24]
CoP/PCNF ^2^-0.4	1 M KOH	138	81	Not reported	[25]
CoP/PCNF-0.4	0.5 M H_2_SO_4_	83	62	Not reported	[25]
Co_2_P/CNF	1 M KOH	275	Not reported	Not reported	[26]
Co_2_P/CNF	0.5 M H_2_SO_4_	241	50	Not reported	[26]
Co_2_P/NPPC ^3^	1 M KOH	125	79	1.00	[30]
CoP@NCNF ^4^	1 M KOH	166	76	Not reported	[31]
MoP/NPG ^5^	1 M KOH	126	56	0.28	[32]
Ni_2_P NA/NF ^6^	1 M KOH	120	37	Not reported	[33]
FeP@NPC	1 M KOH	150	120	0.63	[34]
FeP/NF	1 M KOH	122	20	Not reported	[35]

^1^ N-doped porous CNFs; ^2^ Porous CNFs; ^3^ N and P-co-doped porous carbon composite; ^4^ N-doped carbon nanoflowers; ^5^ N- and P-co-doped graphite carbon nanosheets; ^6^ Nanorod arrays grown on 3D porous nickel foam.

## Data Availability

The data presented in this study are available upon request from the corresponding author.
